# Earlier diagnosis in anorexia nervosa: better watch growth charts!

**DOI:** 10.1186/s40337-020-00321-4

**Published:** 2020-09-03

**Authors:** Morgane Marion, Sylvie Lacroix, Marylène Caquard, Laurence Dreno, Pauline Scherdel, Christèle Gras Le Guen, Emmanuelle Caldagues, Elise Launay

**Affiliations:** 1 CHU de Nantes, Department of adolescent medicine, Pédiatrie générale, 7 quai Moncousu, 44000 Nantes, France; 2 CHU de Nantes Department of child psychiatry, University of Hospital Nantes, Nantes, France; 3 CHU de Nantes, Centre d’Investigation Clinique (CIC004), Nantes, France; 4 CHU de Nantes, Department of Pediatrics, Nantes, France

**Keywords:** Adolescent, Anorexia nervosa., Eating disorder., Feeding disorder., Time to diagnosis., Early diagnosis., Growth charts.

## Abstract

**Background:**

A better understanding of the healthcare pathway of children and adolescents with anorexia nervosa (AN) may contribute to earlier detection and better disease management. Here we measured and compared the symptomatic time to diagnosis (TTD) (time between the first symptoms, as reported by parents, and the diagnosis) and the auxological TTD (time between the deviation in the weight growth curve and the diagnosis).

**Methods:**

We performed a monocentric retrospective study including all patients age 9 years to 16 years who were hospitalized in Nantes University Hospital for AN between 2013 and 2016. We analysed the two TTDs by medical record review and growth curve investigation. TTDs were described by medians and Kaplan-Meier curves. Two profiles of patients were compared according to the kinetics of growth deviation and the occurrence of symptoms.

**Results:**

Among the 137 patients included, the median symptomatic and auxological TTDs was 7.0 months (IQR: 4.0–12.0) and 7.2 months (IQR: 2.0–18.0). TTDs were significantly different but clinically similar. For 48% of the patients, a deviation in the growth curve could have been noted at a median of 9.7 months (IQR: 3.0–18.0) before the first symptoms were reported by parents. Those patients showed significantly slower weight loss than did patients with first symptoms reported before growth deviation (weight loss rate 0.41% vs 1.90% per month, *p* < 0.0001).

**Conclusions:**

Careful study of growth curves remains an essential step in detecting eating disorders, possibly allowing for earlier detection of the disease in nearly half of these patients.

## Plain English summary

Our study highlights the need for more meticulous analysis of growth charts to detect anorexia nervosa earlier in children and adolescents. Children and adolescents should be weighed and measured regularly and growth curves should be systematically constructed. Any deviation in the growth pathway (weight loss or stagnation) should be controlled and investigated if confirmed.

## Introduction

The eating disorder (ED) anorexia nervosa (AN) is more common among girls than boys, with an estimated prevalence of 0.5 to 1% in adolescent girls [1, 2]. AN is responsible for substantial physical and psychological morbidity [1]. The main acute complication of malnutrition is death, but death can also occur over the long term, mainly because of suicide. Among adults, overall mortality with AN is estimated at about 5% [2].

One of the main somatic chronic complications in the pediatric age is the negative impact mainly on height growth and bone density [3, 4]. In fact, malnutrition during the pubertal growth spurt leads to stunted growth, which may not be caught up afterwards [5]. The major consequence is bone demineralization, which has multifactorial pathophysiologic causes (malnutrition, excessive exercise, hypo-estrogenism, hypogonadism with primary and secondary amenorrhoea) [6, 7]. Most of the time, anorexia occurs during adolescence around the menarche, between age 11 to 14 years for most girls. During this perimenarchal phase, 40 to 60% of bone density develops [8, 9]. Having an eating disorder during this critical phase for developing peak bone mass can have long-term implications [4].

The time to diagnosis (TTD), usually defined as the interval between the first symptoms and diagnosis, and its impact on prognosis are widely studied in paediatrics, particularly in pediatric oncology [10, 11]. Our clinical experience with patients with an ED led us to distinguish 2 different diagnostic processes. On one hand, major and fast weight loss in some adolescents will lead to an immediate alert in their family circle and quick access to treatment. On the other, some adolescents will have a slower disease course without or with slight weight loss but will show a deviation in their growth curve, which may lead to less of an alert in their family and longer TTD [12]. We hypothesize that these 2 types of patients have a differing TTD with differing determinants, which we need to consider in future research on the impact of TTD [13].

Studies of AN have included frequent reports of the positive impact of early treatments, particularly on weight restoration trajectories and risk of chronic disease [14–18]. In 2011, Forman et al. looked at predictors of weight outcomes at 1 year depending on the care program [18]. The authors showed that shorter duration of illness before intake and higher percentage of median body weight predicted improved weight outcomes at 1 year. In their study, the average duration of illness before intake in adolescents and young adults was 5.7 to 18.6 months depending on the center. The authors referred to the duration of illness before intake but did not define the TTD, as we propose. Some other studies showed that the median duration of illness before intake was 9 months (IQR: 5.0–16.0) [17] or 1.3 years (IQR: 0.7–4.1) in academic centers and 1.2 years (IQR: 0.5–2.0) in community hospitals [19]. However, these studies also did not clearly define the TTD and did not study the determinants of this delay. Regardless, the definition of TTD and considering the factors that could affect this delay (including the phenotype of the disease) are an important prior step for studying the frequently complex relation between TTD and outcome for a patient.

Our main objective was to describe and compare the symptomatic TTD and auxological TTD in children and adolescents with AN. Our secondary objective was to determine factors associated with the TTDs.

## Methods

### Study design

We conducted a monocentric and retrospective study including all children and adolescents aged 9 to 16 years old who were hospitalized in the day-hospital or had regular full-time hospitalization at Nantes University Hospital from January 2013 to December 2016. The protocol was approved by the local ethics committee of Nantes University Hospital. We relied on the REporting Studies on Time to diagnosis (REST) reporting guidelines to report this study [20].

### Setting

Children and adolescents with AN in the 2 studied administrative French districts (Loire-Atlantique and Vendée) are mainly seen in Nantes University Hospital but can also consult in 2 pediatric psychiatry wards and 2 pediatric wards outside Nantes University Hospital. Adults and adolescents older than 16 years are usually seen in an adult ward (psychiatry or endocrinology) inside or outside Nantes University Hospital.

Patients with AN receiving treatment in Nantes University Hospital are usually referred by a general practitioner or pediatrician or, for children who are severely undernourished, are admitted to the emergency department at Nantes University Hospital. The care pathway for patients with AN is organized around a first step of consultation in the adolescent clinic, then hospitalization in the pediatric ward for diagnostic work-up and re-nutrition according to French recommendations [21]. The criteria for hospitalization of our patients also refers to the French national health authority recommendations (HAS) [21]. The recommendations consider somatic (e.g., a loss of > 2 kg per week, body mass index [BMI] < 13.2 kg/m^2^ at age 15 years and 16 years or BMI < 12.7 kg/m^2^ at age 13 years or 14 years, aphagia, dehydration, syncope, hypothermia, the existence of hemodynamic or electrolyte disorders, electrocardiography abnormalities), psychiatric (e.g., suicidal risk, co-morbidities) and environmental criteria. These criteria are close to those cited in an update of the medical management of eating disorders in adolescents by Golden et al. (2014) [22]. After hospital discharge, patients are followed in the adolescent clinic by adolescent medicine specialists and pediatric psychiatrists as outpatients or in the day-hospital depending on the disease severity. The proportion of patients who are followed at Nantes University Hospital adolescent clinics for ED but are never hospitalized is low, about 5%.

### Participants

Patients were identified in an administrative database with the discharge diagnosis F50.0 according to the *International Classification of Diseases*, 10th revision. The diagnosis of AN was established after the first consultation in the adolescent clinic by using the *Diagnostic and Statistical Manual of Mental Disorders*, Fifth Edition (DSM-5), by 2 specialist physicians (pediatrician and pediatric psychiatrists) jointly caring for the patient. We included only patients treated as inpatients in Nantes University Hospital. We excluded patients who were hospitalized for a very short time but were not followed up at Nantes University Hospital.

### Data collection

Data were collected on patients’ age, puberty status, lifestyle (urban or rural residence, family composition, siblings), family history of ED, disease characteristics (date and age at diagnosis, weight loss at diagnosis, number and duration of hospitalizations) and patients’ growth (weight, height and BMI expressed in standard deviation [SD] units) after age 8 years and up to 2 years after the diagnosis.

### Outcome definition and measures

The auxological TTD was defined as the duration in months from the age at which the weight growth deviated by ≥0.5 SDs from the usual growth curve of the patient to the age at the first consultation in the adolescent clinic. The auxological TTD was obtained from studying the individual growth curve for each patient, which allowed for defining an age before growth curve deviation (last weight before the deviation from the curve) and an age at deviation (first weight deviating from the previous growth path) (Additional file 1). One of the authors (MM) determined the age at the weight-growth deviation by studying the patient’s growth curve as follows: visual tracking, then checking by using the weight data and the corresponding SD for their age according to French growth charts routinely used in France at the time of the study [23]. Hence, the auxological TTD was obtained by calculating the age at first consultation at the adolescent clinic minus the age at weight-growth deviation.

The symptomatic TTD was defined by the duration in months from the age at which the first symptoms occurred (food restriction or sorting and/or change in behaviour) to the age at first consultation in the adolescent clinic. The question about first symptoms was routinely asked during the first interview with the patient and their family, and the response was noted in the medical file and considered the beginning of the symptoms. The symptomatic TTD was then obtained from the consultation reports, as the interval between the age at which parents and/or patients reported the occurrence of first symptoms and the age at first consultation at the adolescent clinic.

Then we compared the auxological and symptomatic TTDs and defined 2 diagnostic profiles of patients: those whose weight-growth deviation appeared before the first symptoms (longer auxological TTD than symptomatic TTD) and those whose weight-growth deviation appeared after the first symptoms (shorter auxological TTD than symptomatic TTD). We divided the patients into 2 groups — those with shorter symptomatic than auxological TTD and those with shorter auxological than symptomatic TTD — to investigate the determinants of delay.

We also calculated the interval from the age at which the first symptoms were noted to the age at weight-growth deviation and the interval from the age at the last consultation before weight-growth deviation to the age at weight-growth deviation (reflecting the interval between 2 weight measurements).

### Statistical analysis

The characteristics of the population are described with median (interquartile range [IQR]) for quantitative data and frequency (%) with 95% confidence intervals (CIs) for categorical data. The auxological and symptomatic TTDs were represented by Kaplan-Meier curve and compared by log-rank test as appropriate for paired samples. Median auxological and symptomatic TTDs were also compared by Wilcoxon test for paired samples. To study the determinants of the auxological and symptomatic TTDs, we tested the association between the TTDs and rural or urban residence (according to the definition of the French national institute of statistics, a town has > 2500 inhabitants), parental situation (living together or separated), ED type (pubertal girls, prepubertal girls, boys), family history of ED, age at diagnosis, and weight-loss rate expressed as a percentage (percentage of total weight loss divided by duration of the weight loss in months). Factors associated with auxological and symptomatic TTD were independently analysed by a Cox proportional-hazards regression model including variables with *p* < 0.2 on univariate analysis. For these analyses, the deviance to linearity was tested (i.e., the relation between TTD and candidate variables) and the delay was transformed into a polynomial variable and classified by quartiles if there was deviation. We also compared the auxological and symptomatic TTDs and the characteristics between the 2 diagnostic profiles of patients (i.e., those with longer auxological than symptomatic TTD with reference to those with shorter auxological than symptomatic TTD). Factors associated with the diagnostic profile with *p <* 0.2 on univariate analysis were entered in the multivariate logistic regression analysis. Hazard ratios, odds ratios (ORs) and 95% CIs were estimated. *P* < 0.05 was considered statistically significant. Data were analysed by using Stata v11.

## Results

### Study population

**Fig. 1:**
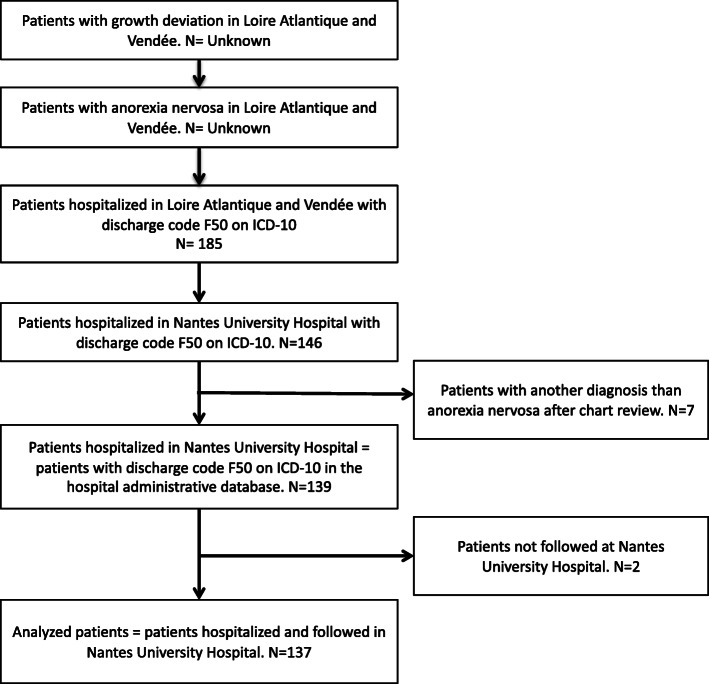
Flowchart of the participants in the study according to the REporting Studies on Time to diagnosis (REST) reporting guidelines. ICD-10, *International Classification of Diseases*, 10th revision

We identified 146 patients hospitalized in Nantes University Hospital with a discharge code for AN in the medical record. For 7 patients, the diagnosis of AN was not confirmed in the medical record. In all, 139 patients were hospitalized for AN in the pediatric ward or in the pediatric day-hospital at Nantes University Hospital between January 2013 and December 2016. Two patients with a follow-up in another center were excluded (Fig. 1).

Among the 137 included patients were 88.3% girls (95% CI: 82.9–93.7) and 11.7% boys (95% CI: 6.3–17.1); the median age at diagnosis was 14 years (IQR: 12.6–14.8) (Table 1). Less than half of the families had 2 children [35% (95% CI: 27.0–43.0)]. The median weight loss at diagnosis was 8.0 kg (IQR: 4.0–11.0), which corresponds to 16.7% of total body mass. The maximum weight loss was 38 kg (52.7% of total body mass). Most patients (95%; 95% CI: 91.4–98.6) were hospitalized in the pediatric ward before receiving treatment in a day-hospital. The median time between the 2 weight measurements allowing to detect an absolute deviation of ≥0.5 SDs in the weight-growth curve was 9 months (IQR: 6.0–12.0). The median weight loss between the two weight measurements was 1.3 SDs (IQR: 0.9–2.0).

**Table 1:** Characteristics of patients with anorexia nervosa who were hospitalized in Nantes University Hospital between 2013 and 2016 (*n* = 137)

	Number of patients	Percentage (95% CI)
**Sex**		
Girls	121	88.3 (82.9–93.7)
Boys	16	11.7 (6.3–17.1)
**Girls puberty status**		
Prepubertal girls	15	12,4 (6,5–18.3)
Pubertal girls	106	87.6 (81.7–93.5)
**Residence**		
Urban	126	92.0 (87.5–96.5)
Rural	11	8.0 (3.5–12.5)
**Parental situation**		
Together	111	81.0 (74.4–87.6)
Separated	26	19.0 (12.4–25.6)
A deceased parent	6	4.4 (1.0–7.8)
**Family history of ED**		
Yes	33	24.1 (16.9–31.3)
No	104	75.9 (68.7–83.1)
**Obesity before AN (BMI > 2 SD)**		
Yes	15	10.9 (5.7–16.1)
No	122	89.1 (83.9–94.3)
**Associated bulimia**		
Yes	20	14.6 (8.7–20.5)
No	117	85.4 (79.5–91.3)
	**Median**	**IQR**
**Age at diagnosis (years)**	14.0	12.6–14.8
Girls	14.1	12.6–14.9
Boys	13.2	12.7–14.1
**Weight loss at diagnosis (kg)**	8.0	4.0–11.0
Girls	8.0	5.0–12.0
Boys	4.8	1.0–6.5
**Weight loss at diagnosis (%)**	16.7	9.8–24.0
Girls	17.8	10.6–24.4
Boys	10.6	4.4–15.4
**Highest BMI (SD)**	0.3	−0.6–1.2
Girls	0.4	−0.4–1.2
Boys	0.0	−1.0–0.8
**Lowest BMI (SD)**	−2.3	−3.1– − 1.5
Girls	−2.2	−2.9– − 1.5
Boys	−3.1	− 3.2– − 1,9

*AN* anorexia nervosa, *BMI* body mass index, *CI* confidence interval, *ED* eating disorder, *IQR* interquartile range, *SD* standard deviation

Half of the patients were hospitalized only once and 23% twice. The median duration of the first hospitalization was 44 days (IQR: 15–73). A weight gain contract was proposed to 67% (95% CI: 58.9–75.1) of patients during their first hospitalization. The median weight gain established in the contracts was 4.5 kg.

### Description, comparison and determinants of TTDs

The median auxological TTD was 7.2 months (IQR: 2.0–18.0) and the median symptomatic TTD was 7.0 months (IQR: 4.0–12.0). The median auxological and symptomatic TTDs significantly differed (*p* = 0.02), but the probability of a diagnosis of AN did not significantly differ between the two TTDs (*p* = 0.90) (Additional file 2). On univariate analysis, we did not find any association between the symptomatic or auxological TTD and ED demographics by sex, parental situation, urban or rural residence, family history of ED, age at diagnosis, current BMI and weight loss rate (Table 2). TTDs (auxological and symptomatic) seemed longer in boys than girls, but probably owing to the small number of boys in our study, the results were not significant. The median auxological TTD was 9.2 months (IQR: 3.0–23.0) for boys and 6.6 months (IQR: 1.9–18.0) for girls (*p* = 0.49). Also, the median symptomatic TTD was 8.5 months (IQR: 3.5–12) and 7.0 months (IQR: 5–10), respectively (*p* = 0.55). On multivariate analysis, faster weight loss was associated with short TTD (i.e., greater risk of instantaneous diagnosis), regardless of type of TTD.

**Table 2:** Univariate and multivariate analysis of factors associated with the auxological and symptomatic time to diagnosis (TTD)

	Auxological TTD						Symptomatic TTD					
	Univariate analysis			Multivariate analysis			Univariate analysis			Multivariate analysis		
	HR	95% CI	p	aHR	95% CI	p	HR	95% CI	p	aHR	95% CI	p
**ED demographic type**												
Pubertal girls	1	–	–				1	–	–	1	–	–
Boys	0.8	0.5–1.5	0.5				0.6	0.4–1.1	0.1	0.7	0.4–1.3	0.3
Prepubertal girls	0.7	0.4–1.3	0.3				0.6	0.4–1.1	0.1	0.7	0.4–1.3	0.3
**Parental situation**												
Together	1	–	–				1	–	–			
Separated	0.9	0.6–1.6	0.9				0.8	0.5–1.3	0.3			
**Residence**												
Urban	1	–	–									
Rural	1.0	0.5–1.9	0.9									
**Family history of ED**												
No	1	–	–	1	–	–	1	–	–			
Yes	1.4	0.9–1.3	0.1	1.2	0.8–1.9	0.4	1.1	0.8–1.7	0.6			
**Age at diagnosis (years)**	0.9	0.8–1.1	0.4				0.9	0.9–1.1	0.7			
**Current BMI (kg/m2)**	0.9	0.8–1.1	0.3				0.9	0.9–1.1	0.9			
**Weight loss rate (% of body mass/month)**	1.9	1.7–2.2	< 0.001	1.9	1.6–2.2	< 0.001	1.2	1.1–1.3	< 0.001	1.2	1.1–1.3	< 0.001

*BMI* body mass index, *ED* eating disorder, *HR* hazard ratio, *aHR* adjusted hazard ratio, *CI* confidence interval

### Comparison of groups of patients by diagnostic profile

**Fig. 2:**
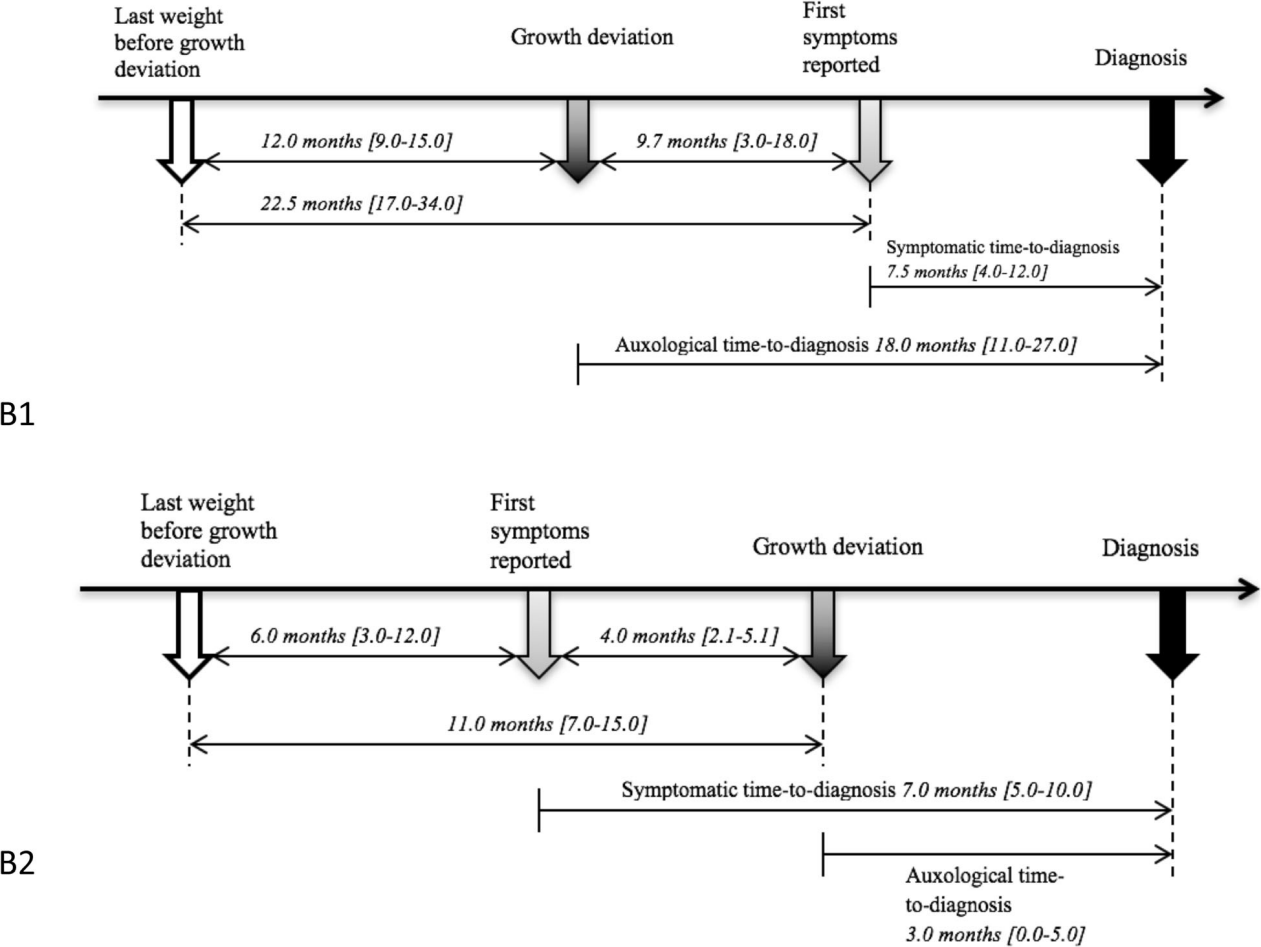
B1) Schema of the diagnostic process for patients with the earliest auxological time to diagnosis (*N* = 66); B2) Schema of the diagnostic process for patients with the earliest symptomatic time to diagnosis (*N* = 71). Data are mean (interquartile range)

Overall, 48% of patients had a growth deviation from the curve, with a median lapse time of 9.7 months (IQR: 3.0–18.0) before the first symptoms were noted by parents or patients (Fig. 2 B1). In this subgroup, patients had been weighed at a median lapse time of 12 months (IQR: 9.0–15.0) before growth deviation was noted (Fig. 2 B1). For the other subgroup (52% of patients), the first symptoms were noted at a median of 4 months [IQR 2.1–5.1] before the growth deviation appeared on growth charts (Fig. 2 B2). In this subgroup, patients had been weighed at a median lapse time of 11 months (IQR: 7.0–15.0) before growth deviation was noted. Symptomatic TTDs did not significantly differ between the 2 subgroups: 7.5 months (IQR: 4.0–10.0) with growth deviation detectable before symptoms were reported versus 7 months (IQR: 5.0–10.0) with growth deviation detectable after the first symptoms were reported (*p* = 0.60). Auxological TTDs significantly differed between the 2 groups: 18 months (IQR: 11.0–27.0) versus 3 months (IQR: 0–5) (*p* < 0.001).

On univariate analysis, weight loss rate was faster for patients with first symptoms reported by parents before the growth curve deviation than those with first symptoms identified after the growth curve deviation (1.9% vs 0.4% per month; OR 0.09 [95% CI: 0.04–0.2]) (*p* < 0.0001) (Table 3). The symptoms were noticed more frequently after the growth curve deviation if the patients were boys or prepubertal girls versus pubertal girls and to a lesser extent if parents were separated. On multivariate analysis, faster weight loss rate and parents living together were significantly associated with noticing the first symptoms before the growth-curve deviation.

**Table 3:** Comparison of patients by diagnostic profile on univariate and multivariate analysis

	Growth curve deviation before first symptoms ^a^ *N* = 66	Growth curve deviation after first symptoms *N* = 71	Univariate analysis^a^			Multivariate analysis^a^		
			OR	95% CI	p	aOR	95% CI	p
**ED demographic type**								
Pubertal girls	45 (68.2%)	61 (85.9%)	1.0	–	–	1.0	–	–
Prepubertal girls	10 (15.2%)	5 (7.1%)	2.7	0.9–8.7	0.08	0.6	0.1–2.6	0.5
Boys	11 (16.6%)	5 (7.1%)	2.9	0.9–9.4	0.05	1.8	0.2–13.3	0.6
**Parental situation**								
Together	50 (75.8%)	61 (85.9%)	0.5	0.2–1.2	0.1	0.2	0.04–0.8	0.03
Separated	16 (24.2%)	10 (14.1%)	1.0	–	–	1.0	–	–
**Residence**								
Rural	8 (12.1%)	3 (4.2%)	3.1	0.8–12.6	0.09	2.5	0.3–21.8	0.4
Urban	58 (87.9%)	68 (95.8%)	1.0	–	–	1.0	–	–
**Family history of ED**								
Yes	15 (22.7%)	18 (25.4%)	0.9	0.4–1.9	0.7			
No	51 (77.3%)	53 (74.6%)	1.0	–	–			
**Age at diagnosis (years), median (IQR)**	14.0 (12.7–14.8)	14.0 (12.5–14.9)	0.9	0.8–1.1	0.5			
**Current BMI (kg/m**^**2**^**) (SD), median (IQR)**	0.3 (−0.7–1.1)	0.4 (−0.2–1.1)	0.9	0.7–1.2	0.5			
**Weight loss rate (% body mass/month), median (IQR)**	0.4 (0.2–0.8)	1.90 (1.1–3)	0.09	0.04–0.2	< 0.0001	0.07	0.03–0.2	< 0.0001

^**a**^ Reference group: Patients with growth curve deviation before first symptoms, i.e., longer auxological TTD than symptomatic TTD

*BMI* body mass index, *ED* eating disorder, *OR* odds ratio, *aOR* adjusted odds ratio, *CI* confidence interval

## Discussion

### Main results and interpretation

In this first study focused on the TTD of AN in children and adolescents, the median symptomatic and auxological TTDs were significantly different but clinically similar: 7.0 and 7.2 months, respectively. The immediate probability of a diagnosis of AN did not differ between auxological and symptomatic criteria. However, for 48% of our patients, the growth-curve deviation was detectable at a median of 9.7 months before the first symptoms occurred. Therefore, the diagnosis could have been established earlier in nearly half of our cases. Fast weight loss rate appeared to be a key factor affecting the TTD and was associated with short auxological and symptomatic TTD.

This study confirmed our clinical impression of 2 different diagnostic processes for patients with AN: 1) patients with “noisy” symptoms and fast weight loss that would alert caregivers and result in an early diagnosis and 2) patients with insidious weight stagnation, whose symptoms remain unnoticed for longer. Symptoms triggered a diagnosis, as opposed to growth-chart deviation leading to an avoidable long time of weight-growth stagnation in half of our patients. In fact, symptomatic TTD was about 7.0 months in both groups (those with symptoms noticed after growth deviation and those with symptoms noticed before growth deviation), whereas auxological TTD was much longer when symptoms were noticed after growth deviation was detectable on growth charts (18 vs 3 months). Our hypothesis is that parents alerted by symptoms brought their children to a medical consultation, during which weight stagnation or loss was then interpreted as pathological, whereas a deviation on growth deviation noted before symptoms were reported by parents did not lead to a systematic search for these symptoms and was not interpreted as pathological by clinicians. Moreover, patients were weighed in both groups at a comparable median interval of 11 and 12 months before weight growth deviation was detectable. The diagnostic delay was then due to suboptimal analysis of the growth chart by physicians and not lack of consultation or delay in seeking care by parents.

The symptoms were identified more often after the growth curve deviation in prepubertal versus pubertal girls. This observation is consistent with the literature, in which younger patients are described to more frequently have unusual clinical presentations than older youths. Instead of a fast weight loss rate, they may present a weight stagnation or an inability to gain weight. Moreover, dysmorphophobia is often more undetectable in the first stage for younger than older adolescents. These atypical presentations may be responsible for a late diagnosis [16, 24].

We did not find any sex differences in TTDs. In contrast to the study of Strandjord et al. [25], TTD (symptomatic and auxological) was longer for the boys than girls in our cohort but was not significant perhaps because of the low number of boys.

### Internal validity

To establish a definition of the TTD, we referred to the well-known diagnostic triad of AN: anorexia, weight loss, amenorrhoea [2]. For evaluating symptomatic TTD, we used symptoms of food restriction and sorting detected by parents and patients as reported at the first consultation at the adolescent clinic. However, the real duration of symptom evolution may be underestimated by this method. Indeed, a 2014 study showed that parents frequently attributed the early signs of AN to the normal development of adolescents and expected the weight loss to be short-lasting, which delayed the first medical consultation [26]. We did not retain the amenorrhoea criterion to study TTD. Indeed, this piece of data was not relevant in our population composed partly of boys and prepubertal girls and was shown to be invalid in children and adolescent populations [16]. Moreover, in the 2013 DSM-5 update, the presence of amenorrhoea is no longer necessary for a diagnosis of AN [2]. For the symptomatic diagnosis of AN, tools available for primary care are poor. Only the SCOFF questionnaire is available, but it was validated in French only for an adult population [16, 27].

Auxological TTD was defined by a meticulous analysis of growth charts. The definition of weight growth deviation as ≥0.5 SDs of the usual growth path was arbitrarily chosen and may have overestimated auxological TTD, but, as in other fields of pediatrics, we lack consensus on defining the abnormal growth curve for early detection of AN [13]. Comparison with growth charts of “safe” controls may be helpful to test the specificity of this weight-growth deviation.

### Strengths and limitations

We present detailed data and auxological characteristics of a large population of children and adolescents with AN. Because of the pediatric nature of our hospital, which admits patients under age 16 years only, the population of our study is younger than in studies from other countries and therefore not representative of all patients with AN. However, our study has some limitations, the first of which is recruitment bias. The adolescents followed at Nantes University Hospital, a referral center for ED, probably have more severe somatic and psychiatric presentation with greater weight loss or longer TTD than other adolescents. Besides, the definition method of TTD implies a memory recall bias concerning the symptoms detected by parents, especially due to the denial inherent in AN. Symptoms more detailed than restrictions such as occasionally skipping a meal or discrete dietary changes could not be detected in our study because of the retrospective approach. We would have gained accuracy by using a standardized questionnaire. This could be a great contribution in future studies. Finally, the definition of the diagnosis date is questionable. The choice of the first specialized consultation is pragmatic and corresponds to the beginning of the specific management of the disease. Moreover, confirmation of the diagnosis often occurs after this consultation. However, this choice does not account for previous consultations with the physician for the same reason, nor treatment in primary care, which could have been initiated before the specialized consultation. Because of the retrospective data collection and the occasionally incomplete data in medical files, the choice of the first hospital consultation date ensured an objective and constant definition of TTD among patients.

## Conclusion

Our study highlights the need for a more meticulous analysis of growth curves to detect AN earlier. Children and adolescents should be weighed and measured regularly, and growth curves should be systematically constructed. Any deviation in the growth pathway (weight loss or stagnation) should be controlled and investigated if confirmed. Consequences of the TTD for adolescents with AN have not been studied in a large population. Long TTD and particularly long auxological TTD may have consequences on bone mineralisation and adult size. A multicentric prospective study should be performed to better define auxological TTD and better evaluate its consequences and bone mineralisation [28]. Comparison of growth curves for healthy children and adolescents and those with AN could also help define a screening algorithm.

## Supplementary information

**Additional file 1.** Example of a weight growth curve. Weight deviating by ≥0.5 standard deviations between age 13 and 14.25 years. Age at growth curve deviation: 14.25 years; age at the last weighing before the deviation: 13 years.

**Additional file 2.** Kaplan-Meier curves for auxological and symptomatic time to diagnosis representing the proportion of patients screened by a growth deviation curve or first symptoms.

## Data Availability

The datasets used and/or analysed during the current study are available from the corresponding author on reasonable request.

## Abbreviations

AN: Anorexia nervosa
BMI: Body mass index
CI: Confidence interval
ED: Eating disorder
IQR: Interquartile range
OR: Odds ratio
SD: Standard deviation
TTD: Time to diagnosis
