# Supplementary figures and images for: Earlier diagnosis in anorexia nervosa: better watch growth charts!
Source: J Eat Disord. 2020 Sep 3;8:42. doi: 10.1186/s40337-020-00321-4 (PMC7469097; doi:10.1186/s40337-020-00321-4)

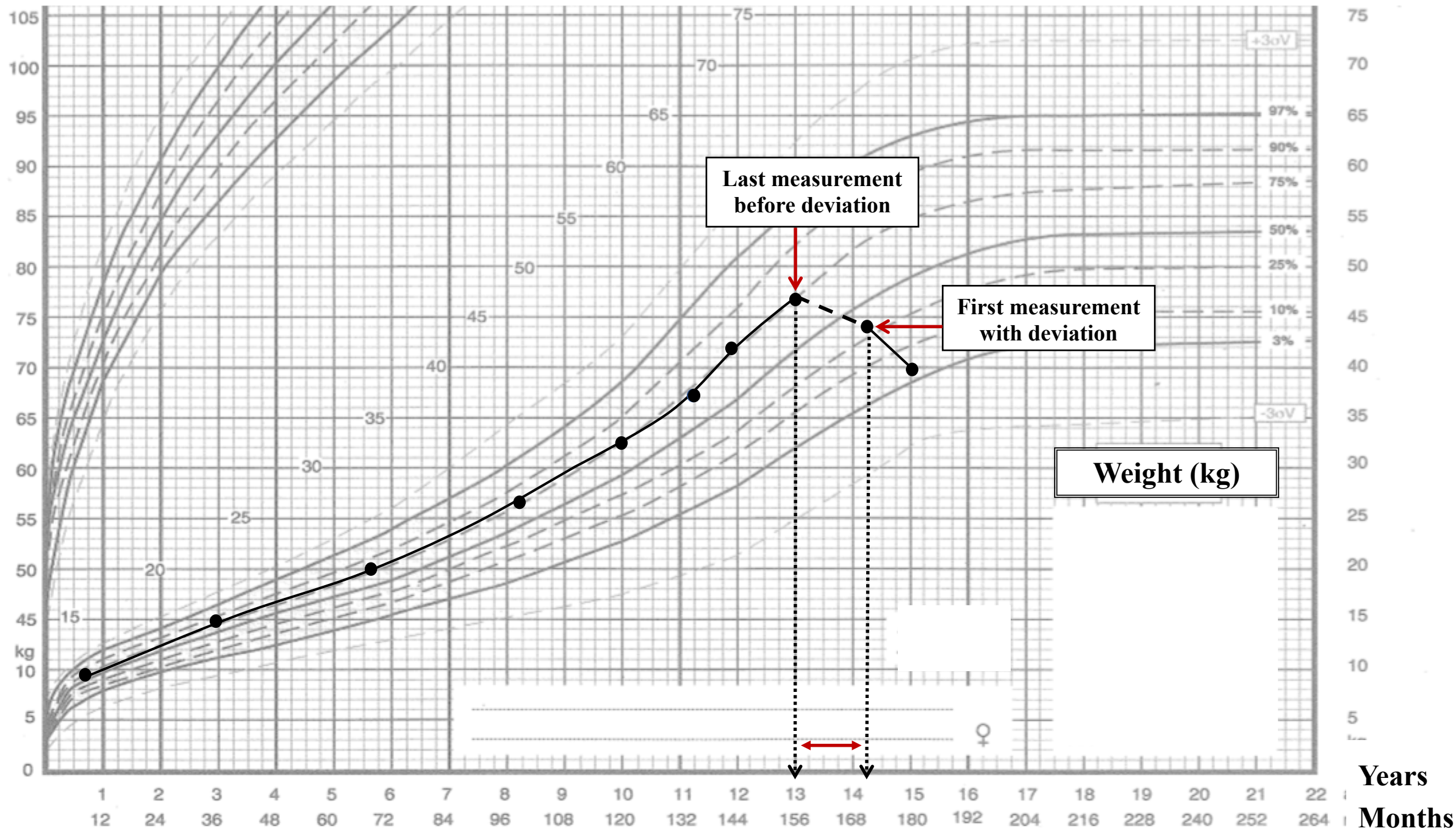

Supplement: Supplementary file 1 — Additional file 1. Example of a weight growth curve. Weight deviating by ≥0.5 standard deviations between age 13 and 14.25 years. Age at growth curve deviation: 14.25 years; age at the last weighing before the deviation: 13 years. [file 40337_2020_321_MOESM1_ESM.pdf]

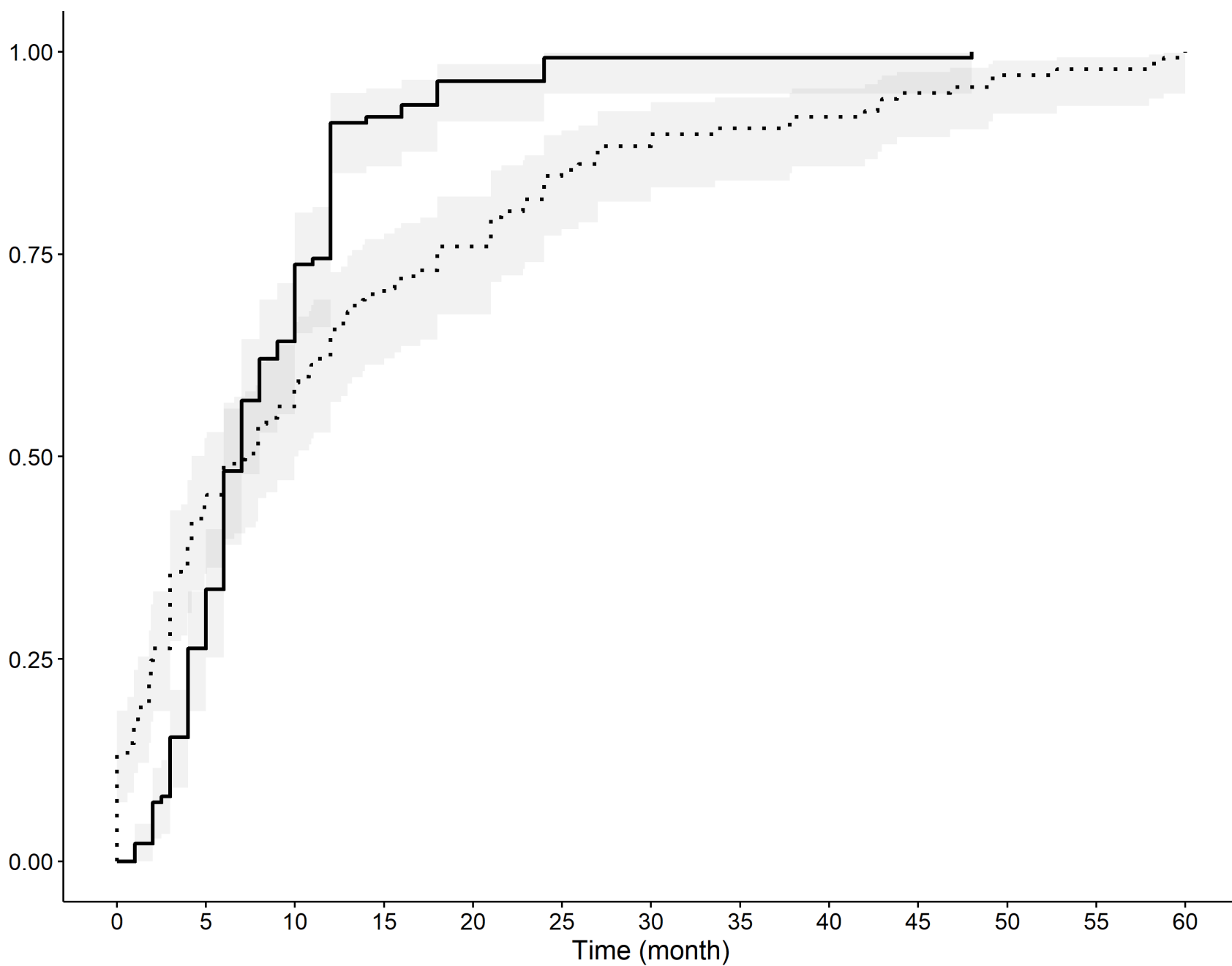

Time To Diagnosis: •• Auxological TTD — Symptomatic TTD

Supplement: Supplementary file 2 — Additional file 2. Kaplan-Meier curves for auxological and symptomatic time to diagnosis representing the proportion of patients screened by a growth deviation curve or first symptoms. [file 40337_2020_321_MOESM2_ESM.pdf]
